# P08 A Phase 1, randomized, double-blind study to evaluate the safety, tolerability, and immunogenicity of a 21-valent pneumococcal conjugate vaccine (PCV) (V116) in adults

**DOI:** 10.1093/jacamr/dlac133.012

**Published:** 2023-01-19

**Authors:** Heather Platt, Doreen Fernsler, Nancy Gallagher, Aditi Sapre, Adam Polis, Lori Hall, Gretchen Tamms, Howard Schwartz, Julie Skinner, Joseph Joyce, Rocio Murphy, Luwy Musey

**Affiliations:** Merck & Co. Inc., Kenilworth, NJ, USA; Merck & Co. Inc., Kenilworth, NJ, USA; Merck & Co. Inc., Kenilworth, NJ, USA; Merck & Co. Inc., Kenilworth, NJ, USA; Merck & Co. Inc., Kenilworth, NJ, USA; Merck & Co. Inc., Kenilworth, NJ, USA; Merck & Co. Inc., Kenilworth, NJ, USA; Research Centers of America, LLC, Hollywood, FL, USA; Merck & Co. Inc., Kenilworth, NJ, USA; Merck & Co. Inc., Kenilworth, NJ, USA; Merck & Co. Inc., Kenilworth, NJ, USA; Merck & Co. Inc., Kenilworth, NJ, USA

## Abstract

**Background:**

V116, an investigational 21-valent PCV, contains the following pneumococcal polysaccharides (PnPs): 3, 6A, 7F, 8, 9N, 10A, 11A, 12F, 15A, 16F, 17F, 19A, 20, 22F, 23A, 23B, 24F, 31, 33F, 35B, and a de-O-acetylated 15B (deOAc15B). This phase 1 study evaluated the safety, tolerability, and immunogenicity of V116 in pneumococcal vaccine-naive adults compared with the 23-valent polysaccharide pneumococcal vaccine (PPSV23).

**Methods:**

Adults (*n*=90) 18–49 years were randomized 1:1:1 to receive a single dose of V116-1 (2 μg dose/each PnPS, V116-2 (4 μg dose/PnPS) or PPSV23. Adverse events (AEs) were collected following vaccination. Pneumococcal serotype-specific opsonophagocytic activity (OPA) was measured prior to and 30 days postvaccination (Day 30).

**Results:**

There were no serious AEs, deaths, or discontinuations due to AEs. Immune responses at Day 30 in the V116-1 and V116-2 groups were generally comparable to PPSV23 for the common serotypes and higher than PPSV23 for the unique serotypes. At Day 30, the OPA GMTs were higher in the V116-2 group compared with the V116-1 group for all serotypes except 9N. The OPA geometric mean titre ratio (95% CI) (V116-2/PPSV23) ranged from 0.89 (0.58, 3.51) to 2.40 (1.24, 4.62) for all common serotypes and 2.80 (1.64, 4.79) to 58.07 (25.10, 134.33) for all unique serotypes; the lower bound of the 95% CI for the OPA GMT ratio (V116-2/PPSV23) was >0.5 for all common serotypes and >1.0 for all unique serotypes.

**Conclusions:**

These safety and immunogenicity data support the continued development of V116 for the prevention of pneumococcal disease in adults.

